# A qualitative journey mapping study of prenatal care experiences among rural pregnant women in Northeast China

**DOI:** 10.3389/fpubh.2026.1787403

**Published:** 2026-04-29

**Authors:** Xiaoxi Luo, Jiayuan Zhang, Nan Yang, Junyu Chen, Xiaolin Gu, Kexin Han, Yuqiu Zhou

**Affiliations:** Harbin Medical University, Daqing, China

**Keywords:** field observation, journey mapping, pregnant women, prenatal health care, qualitative interviews, rural areas

## Abstract

**Background:**

The participation rate of prenatal healthcare in China has increased slowly, and the imbalances and inadequacies in the development of maternal and child health between urban and rural areas are particularly prominent. A key reason for this is that a comprehensive understanding of rural women’s utilization of prenatal healthcare services and their needs has not been fully established, thus failing to fully leverage pregnant women’s agency in prenatal healthcare. The study aimed to identify the multi-dimensional needs of rural women in the process of utilizing prenatal healthcare services through constructing a journey map, so as to optimize the full pregnancy cycle management of this group.

**Methods:**

The study population consists of rural pregnant women who attended obstetric clinics at healthcare institutions in Northeast China from January to May 2024. A qualitative multi-method design was adopted, integrating non-structured field observation and descriptive phenomenological qualitative interviews to comprehensively gather rural women’s emotions, perceptions, experiences, and needs regarding prenatal care services. Field observation provided contextual, behavioral, and environmental insights into service delivery processes and stakeholder interactions, while qualitative interviews delved deeply into the subjective lived experiences, emotional responses, and contextual factors shaping care utilization. The collected data were systematically analyzed and synthesized to construct a detailed journey map that delineates the sequential stages of prenatal care, key touchpoints, behavioral patterns, needs, pain points, and opportunities for improvement. Ethical approval was obtained from the Ethics Committee of Harbin Medical University, Daqing, No. HMUDQ20230330001.

**Results:**

The journey comprises five phases. The first stage marks the initiation of prenatal care services. Pregnant women are often unfamiliar with the procedures and have various concerns and anxieties about their upcoming pregnancy journey. In the second stage, pregnant women begin participating in non-invasive prenatal screening and start focusing on long-term nutrition and gestational weight management. In the third stage, maternal-fetal interaction increases, and ultrasound screenings for congenital anomalies become a critical task. Lifestyle changes and management pose significant challenges during this period. The fourth stage emphasizes proactive prevention and screening for pregnancy-related complications. Guiding pregnant women in self-monitoring is essential, and their needs for postpartum care for both mother and baby gradually increases. In the fifth stage, pregnant women frequently engage with prenatal care services, experiencing both anticipation and anxiety as they prepare for delivery.

**Conclusion:**

This exploratory qualitative multi-method study conducted in rural Northeast China developed a five-stage journey map of prenatal healthcare service utilization. The findings are context-specific to rural Northeast China and do not imply broad generalizability; they highlight key optimization points for prenatal care management for rural pregnant women in similar regional settings.

## 1 Introduction

Prenatal health care is a health service offered by medical and health institutions at all levels from the date of confirmed pregnancy to the time immediately before delivery. It provides health education, consultation and guidance, comprehensive physical examination, obstetric examination and auxiliary examinations for pregnant women at different time nodes. In China, standardized prenatal care is defined as a systematic and structured approach wherein pregnant women receive targeted healthcare services based on the specific stages of pregnancy. This encompasses appropriate health education and guidance tailored to each stage, along with 7 to 11 prenatal check-ups with varying content and focus. For high-risk pregnancies, the frequency and intensity of check-ups should be increased accordingly. Standardized participation in prenatal health care has been proven effective in safeguarding the physical and mental health of both mothers and infants, and reducing the incidence rate of birth defects as well as the cesarean section rate of pregnant women (1, 2). As the largest developing country in the world, China has the most numerous groups of women and children. Maternal and child health, as well as pregnancy and childbirth health, still encounter numerous practical challenges such as unbalanced regional development and inadequate services. The main cause of this phenomenon is that the traditional prenatal health care management mode mostly depends on the passive acceptance of pregnant women, but fails to truly understand the experiences and needs of women in the process of prenatal care. Improving the utilization of prenatal healthcare services for rural women is, thus, a key research focus.

Pregnancy is a unique phase in a woman’s life cycle. During this period, fluctuations in hormone levels and increased functional demands on various organs lead to a range of physical discomforts including nausea, frequent urination, pain, weight changes, and sleep disturbances (3–5). Additionally, pregnant women may experience emotional regulation challenges such as anxiety and depression (6, 7). These factors can adversely affect both maternal and fetal health as well as the overall quality of life. Despite the desire for effective treatment, pregnant women often opt not to address their physical discomfort or rely solely on simple self-care measures due to concerns about the safety of pharmacological interventions and the associated costs. Simultaneously, pregnant women require comprehensive information to ensure their health during pregnancy, facilitate safe delivery, and support scientific child-rearing. Studies indicate that the information demand among pregnant women is as high as 90%, and this demand is equally prominent among rural pregnant women while existing resources are inadequately adapted to their needs (8). However, current information resources predominantly focus on routine topics such as prenatal care, delivery options, benefits of breastfeeding, and newborn care. Less attention is given to sensitive issues like sexual activity during pregnancy and postpartum contraception (9, 10). Additionally, critical areas such as rational drug use during pregnancy, self-monitoring techniques, labor pain management, and emotional regulation are often underrepresented (11–13). On the other hand, current health education methods in prenatal schools and clinics for pregnant women predominantly rely on theoretical lectures and the distribution of written materials and diagrams. This approach leads to suboptimal spatial presentation, timeliness, and reusability of the information. Furthermore, influenced by the traditional ideologies of agricultural societies and prevailing marriage and family norms, rural women in China frequently cohabit with their husband’s family members, leading to frequent intergenerational conflicts. During pregnancy, these women often assume limited and stereotypical roles, resulting in a significant need for external support. Pregnant women strongly desire understanding and support from families, peers, and healthcare providers to enhance their engagement in prenatal care.

Pregnant women are the primary implementers and beneficiaries of maternal health behaviors. Therefore, comprehensively understanding their behaviors, expectations, emotions, and interactions with providers can help improve person-centered prenatal care.

Previous qualitative studies have examined the experiences, barriers and psychological needs of pregnant women in prenatal care. Research in rural China has further identified challenges such as insufficient guidance, uneven resource distribution and limited accessibility of maternal health services (14). Meanwhile, journey mapping has been increasingly used in health services research to visualize care pathways, identify pain points and improve service experiences (15). However, few studies have combined this approach to systematically explore the full prenatal care trajectory among rural pregnant women in China. Patient journeys can also reveal areas where unmet needs exist, identify key stakeholders, and highlight how the management can be improved (16, 17). Therefore, based on existing literature gaps, this study constructs a journey map of prenatal care services for rural pregnant women to identify key touchpoints and pain points, which supplements and enriches the existing research on rural maternal health.

## 2 Methods

### 2.1 Study design

This study adopted a qualitative multi-method design, integrating non-structured field observation and descriptive phenomenological qualitative interviews. This dual-method approach was employed to comprehensively capture the multi-dimensional experiences, perceptions, and unmet needs of rural pregnant women throughout their prenatal healthcare journey. Field observation provided contextual, behavioral, and environmental insights into service delivery processes and stakeholder interactions, while qualitative interviews delved deeply into the subjective lived experiences, emotional responses, and contextual factors shaping care utilization. These complementary data sources enhanced the credibility, depth, and robustness of the study findings, ensuring a holistic understanding of the prenatal care experience for rural women.

### 2.2 Study setting and population

This study was conducted between January and May 2024 in Anda City (Suihua Prefecture) and Daqing City, Heilongjiang Province, China. A total of 6 healthcare institutions were purposively selected to represent diverse levels of prenatal care service capacity and accessibility in rural regions, including 1 municipal-level maternal and child health hospital, 3 county-level maternal and child health hospitals, and 2 township health centers affiliated with the aforementioned municipal/county institutions. These sites were chosen to cover both urban–rural fringe and remote rural areas, ensuring the capture of variability in prenatal care service delivery across different administrative and resource levels. The study population consisted of rural pregnant women receiving prenatal care at the selected institutions, with consistent eligibility criteria for both field observation and qualitative interviews: aged≥18 years; permanent rural residency (Operational definition of rural: refers to women whose household registration is in rural/township areas, and who have lived continuously in rural areas for≥2 years); currently pregnant at the time of recruitment; and able to communicate fluently in Mandarin or local dialect to ensure effective data collection. Exclusion criteria included diagnosed intellectual disabilities, confirmed mental health disorders that may impair cognitive judgment or communication, communication impairments precluding interaction with researchers or healthcare providers, and refusal to participate or inability to provide informed consent. For field observation, all eligible rural pregnant women attending prenatal care during the study period were included as observational subjects. For qualitative interviews, participants were further selected through purposive sampling to ensure diversity in age, educational level, gestational weeks, gravidity, and parity, thereby capturing a broad range of prenatal care experiences.

### 2.3 Fieldwork observation

#### 2.3.1 Observation rationale and design

A non-structured observational approach was utilized to capture the naturalistic context of prenatal care service utilization without imposing on researcher-defined frameworks. This design was chosen to accommodate the variability in service delivery models across different healthcare institutions and to document unanticipated behaviors, environmental factors, and interaction dynamics that may influence the care experience. Unlike structured observation, this approach minimized researcher bias and allowed for the holistic collection of data on the temporal, spatial, and social dimensions of prenatal care engagement.

#### 2.3.2 Data collection procedures

Two trained researchers conducted on-site observations during peak service hours (8:00–11:30 a.m. and 1:30–5:00 p.m.) to ensure representative data on service flow and patient-provider interactions. Prior to data collection, researchers completed standardized training on non-participant observation techniques, ethical conduct, and objective documentation to minimize observer interference. During observations, researchers adopted a passive, non-intrusive role to avoid disrupting the natural flow of care. Data were recorded through: (1) detailed textual field notes (documenting physical environments, patient behaviors, staff-patient communication, procedural steps, and temporal sequences) and (2) schematic diagrams (mapping spatial layouts, patient flow pathways, and key interaction touchpoints). Observations were rotated across sites weekly to ensure diversity in data collection, with a total of 92 h of observation completed (15–16 h per institution, covering Monday to Friday to ensure representation across different weekdays). Inter-observer consistency checks were conducted biweekly: two researchers independently observed the same service encounter, compared notes, and resolved discrepancies through consensus, any discrepancies in records were resolved through in-depth group discussion until consensus was reached, ensuring the reliability and objectiveness of observational data. A total of 136 rural pregnant women were observed during the study period.

#### 2.3.3 Data analysis

Observation data were thematically analyzed using an inductive approach. First, textual notes and diagrams were transcribed and organized chronologically by service stage. Two researchers independently coded the data using NVivo 12 software to identify emergent themes. Codes were then clustered into broader categories and cross-validated against schematic diagrams to ensure alignment with contextual observations. A third senior researcher reviewed the thematic framework to resolve coding discrepancies and ensure analytical rigor. The final thematic framework was used to map the sequential stages of prenatal care and identify critical touchpoints, behavioral patterns, and system-level barriers.

### 2.4 Qualitative interviews

#### 2.4.1 Methodological rationale

A descriptive phenomenological approach, guided by Colaizzi’s seven-step method (18), was employed to explore the subjective lived experiences of rural pregnant women in prenatal care. This methodology was selected for its ability to uncover the implicit meanings, emotional responses, and contextual factors shaping care utilization-dimensions that are often inaccessible through observational methods alone. Colaizzi’s systematic framework ensures rigorous analysis of phenomenological data, enhancing the trustworthiness and transferability of the findings.

#### 2.4.2 Participant recruitment

Purposive sampling was used to recruit participants from the outpatient departments of the selected healthcare institutions. Eligibility criteria were: (1) aged ≥18 years; (2) rural permanent resident (≥2 years of residency); (3) currently pregnant; (4) fluent in Mandarin or local dialect; (5) no cognitive or mental health conditions impairing informed consent or interview participation; and (6) voluntary agreement to participate. Participants were recruited across different age groups, educational levels, trimesters, gravidity, and parity to ensure demographic diversity and capture a broad range of experiences. Recruitment ceased when data saturation was achieved-defined as the point at which three consecutive interviews yielded no new themes or insights. Data saturation was achieved when three consecutive new interviews yielded no additional themes. Saturation was confirmed across all participant subgroups, including different gestational trimesters, age groups, and educational levels, ensuring comprehensive and representative data. A total of 16 participants were enrolled (Table 1), with demographic characteristics reflecting the rural pregnant population in the study regions. The 16 interviewees were distributed across 6 healthcare institutions and included 4 in first trimester, 5 in second trimester, and 7 in third trimester, ensuring coverage of different pregnancy stages.

**Table 1:** General characteristics of the participants.

Characteristics	Pregnant women (*n* = 16)
Age range (years)	19–40
Education level	
Primary school	2
Middle school	8
High school	4
College and higher	2
Gestational weeks	
First trimester	4
Second trimester	5
Third trimester	7
Gravidity	
1	8
2	6
3	2
Parity	
0	10
1	5
2	1

#### 2.4.3 Interview procedures

Semi-structured interviews were conducted by two researchers with expertise in maternal and child health and qualitative research. Interviews were held in a private room at the healthcare institution, based on participant preference, to ensure comfort and privacy. The interview duration ranged from 30 to 60 min, with a median of 45 min. The interview guide (Table 2) was developed based on the study objectives and revised following expert consultation (three maternal and child health specialists and two qualitative research methodologists) to ensure content validity and cultural relevance. The guide explored four core domains: (1) experiences with prenatal care services; (2) emotional responses to care utilization (positive/negative experiences and associated triggers); (3) prioritized needs during pregnancy and unmet service gaps; and (4) recommendations for service improvement. Probing questions were used to elicit rich, contextualized narratives. All interviews were audio-recorded with participant permission and transcribed verbatim within 24 h. Field notes were documented during interviews to capture non-verbal cues and contextual details.

**Table 2:** Outline of the interview.

1. What kind of support and services did the healthcare providers provide you during your pregnancy? At what time and location did you receive prenatal care services? 2. During the process of receiving prenatal care services, what pleasant/unpleasant experiences and feelings did you have? Please give examples. 3. What were your needs during pregnancy? (Please rank them.) Which of your needs were met by the healthcare providers when they provided prenatal care services? And which ones still need to be met? 4. What measures do you think can be taken to improve the current prenatal care services?

#### 2.4.4 Data analysis

We used NVivo 12 to code and manage all interview data. This study adopts the Colaizzi descriptive analysis framework Colaizzi, (18), divided into following analytical steps. (1) Reading of interviews by two researchers who reread transcripts several times to become immersed in the data. (2) Identification of significant statements related to rural pregnant women’s experiences with prenatal care services. (3) Extracting meaningful fragments through team discussions. (4) Organization of each significant statement into meaningful units and subthemes into major themes. (5) Linking themes closely to research phenomena and detailing them. (6) Providing feedback on the results to participants to ensure the authenticity of the content.

#### 2.4.5 Bracketing, reflexivity, and rigor

Given the sensitivity of the topic, we established a participant relationship by listening and using a non-judgmental approach. We ensured that the interview questions were neutral and open-ended to allow participants to share their opinions about their experiences. To describe the experiences of the participants from a research perspective as impartially as possible, we managed to bracket the preconceptions of the phenomenon by adopting a multidisciplinary team approach to interviewing and data analysis. The researchers were female health professionals with experience in maternal health and qualitative research. Prior to data collection, we documented and bracketed our preunderstandings to minimize bias and maintain openness to participants’ perspectives. Regular reflexive discussions were held throughout data collection and analysis to reflect on potential influences of researcher subjectivity. Two authors independently coded the data, and discrepancies were resolved through group discussion until consensus was reached. Member checking was also performed to enhance the credibility of the findings. These measures strengthened the rigor and trustworthiness of the study.

### 2.5 Journey map construction

Data from field observation and qualitative interviews were integrated through a convergent qualitative analysis to identify consistent themes, stages, touchpoints, needs, and pain points. Findings from both data sources were systematically compared and synthesized to ensure the journey map reflected both objective service processes from observation and subjective experiences from interviews. Any discrepancies were discussed and resolved by the research team to ensure analytical consistency.

The prenatal healthcare service journey map was developed using a rigorous, step-by-step analytical workflow:

1. All raw data (field notes and interview transcripts) were independently coded by two researchers using NVivo 12.
2. Initial codes were aggregated into subthemes and main themes related to service processes, behaviors, emotions, needs, and barriers.
3. Themes from observation and interviews were matched, compared, and merged to identify convergent findings.
4. Thematic results were used to iteratively define five sequential stages of the care journey.
5. The stage division and core components (touchpoints, needs, pain points) were validated through cross-checking between two data sources.
6. The preliminary journey map was further reviewed by a multidisciplinary expert panel and verified via member checking with participants to ensure authenticity and accuracy.

Detailed procedures are as follows:

#### 2.5.1 Delineation of prenatal care stages

The entire prenatal healthcare trajectory was first divided into five discrete stages based on the chronological sequence of service utilization identified via field observations. This stage division was cross-validated against thematic analysis results of qualitative interviews to ensure consistency with rural pregnant women’s actual care-seeking experiences, avoiding discrepancies between objective service processes and subjective perceptions.

#### 2.5.2 Extraction of core components

For each stage, core components were extracted through integrated analysis of observational and interview data:

Touchpoints: Specific interaction loci and scenarios of service engagement, identified from field observation records and supplemented by interview narratives.
Behaviors: Behavioral patterns and care-seeking actions, synthesized from field observation data and interview accounts.
Needs: Multi-dimensional needs (physical, emotional, informational, social), compiled from interview thematic analysis and field-documented unmet needs.
Pain Points: Systemic barriers and adverse experiences, extracted via convergent analysis of observational data and self-reported negative experiences in interviews.
Opportunities for Improvement: Targeted intervention strategies, formulated based on participant recommendations from interviews and system-level bottleneck analysis from field observations.

#### 2.5.3 Visual integration and mapping

Using the extracted components, a visual journey map was constructed to present the five-stage trajectory in logical sequence. Adopting a horizontal layout with chronologically labeled stages, corresponding components were organized into parallel thematic modules to intuitively reflect their interrelationships. Emotional responses throughout the journey were integrated via standardized iconography or color-coding to dynamically visualize emotional fluctuations across phases.

#### 2.5.4 Validation and revision

The preliminary map underwent rigorous peer review by a multi-disciplinary panel to assess accuracy, comprehensiveness, and logical coherence. Purposively selected participants conducted member checking to verify alignment with personal care-seeking experiences and needs. Based on consolidated feedback, iterative revisions were made to refine descriptions, optimize layout, and supplement omitted information, ensuring the final map authentically and comprehensively captures rural women’s multi-dimensional experiences and needs in prenatal care. To ensure transparency, an example of data integration is provided. In Stage 1 (Establishing Medical Records), field observations showed that many women were confused by unclear signage and long waiting times. Interview data further supported this finding:“I did not know where to go for the first check-up and walked back and forth many times” (Participant 1). These combined data were used to identify the pain point of unclear service processes in the journey map.

### 2.6 Ethical considerations

The research was approved by the Ethics Committee of Harbin Medical University (Daqing), Number: HMUDQ20230330001, dated March 30, 2023. Prior to recruitment, all potential participants were provided with a written informed consent form explaining the study purpose, procedures, potential risks (minimal), benefits, and their right to withdraw at any time without penalty. Anonymity was ensured by replacing all identifying information with pseudonyms; confidentiality was maintained through password-protected storage of audio recordings and transcripts, with data de-identified prior to analysis. Verbal and written consent was obtained from all participants before interviews commenced.

## 3 Results

### 3.1 Fieldwork observation

Rural women’s prenatal healthcare service can be categorized into five distinct stages: establishing medical records for pregnant women, non-invasive prenatal screening, ultrasound screening, screening for complications, and fetal monitoring/prenatal preparation. Table 3 presents a synthesis of key observations regarding service experiences and the involved participants across these stages. For a detailed breakdown of the specific settings, spatial–temporal interactions, and comprehensive activities within each stage, please refer to Supplementary Table S1.

**Table 3:** Key observations and participants involved in prenatal care stages for rural women.

Stage	Participants	Key observations
Establishing medical records for pregnant women	Pregnant women Family members Obstetric doctor Ultrasound physician Obstetric nurse Phlebotomy nurse	1. Unclear signage and electronic display information led to widespread confusion about registration departments and examination room locations among most observed pregnant women 2. Lack of guidance on the sequence of multiple examinations, resulting in unnecessary back-and-forth between departments 3. Prolonged waiting time for consultation (average 32 min) causing visible anxiety
Non-invasive prenatal testing	Pregnant women Family members Obstetric doctor Phlebotomy nurse	1. Limited verbal explanation of NIPT principles and clinical significance, leading to confusion among pregnant women with primary or middle school education; 2. Obvious anxiety was commonly observed among women during the 2-week waiting period for results 3. No clear notification mechanism for result collection, requiring repeated on-site inquiries by some pregnant women
Ultrasound screening	Pregnant women Family members Obstetric doctor Ultrasound physician Obstetric nurse	1. Lack of pre-examination guidance on cooperation methods, leading to some women requiring repeated examinations or rescheduling 2. Long waiting time for ultrasound (average 40 min) with no estimated waiting time provided 3. Music playback system volume too low to relieve anxiety, and limited variety of provided snacks for women with pregnancy-induced hunger
Screening for complications	Pregnant women Family members Obstetric doctor Obstetric nurse Phlebotomy nurse	1. Unpalatable taste of concentrated glucose solution, causing nausea and vomiting in 5 observed cases 2. No supplementary food provided during the 2-h waiting period, leading to obvious hunger and fatigue 3. Lack of guidance on self-monitoring of blood pressure and blood glucose after examination, with most women unaware of normal reference ranges
Fetal monitoring/prenatal preparation	Pregnant women Family members Obstetric doctor Obstetric nurse	1. Uncomfortable monitoring belts and prolonged monitoring time leading to body soreness in third-trimester pregnant women 2. Lack of on-site guidance on recognizing preterm labor signs and emergency response measures 3. Insufficient information on delivery preparation provided during consultation

### 3.2 Qualitative interviews

A total of 16 pregnant women in rural China were interviewed in this study. The characteristics of the participants are shown in Table 1.

In interviews, pregnant women generally reported relative satisfaction with several aspects of current prenatal care services, including the professional explanations and appropriate reassurance provided by healthcare providers, as well as the protection of their privacy during various free prenatal examinations. The main points of dissatisfaction among pregnant women include prolonged waiting times for medical consultations, insufficient time for effective communication with healthcare providers, and inadequate promotion of the hospital’s prenatal education programs, which limits their ability to fully utilize this valuable educational resource.

Furthermore, pregnant women have diverse needs at different stages of pregnancy, such as the dissemination of favorable national childbirth policies, family accompaniment, access to pregnancy-related information, weight management, dietary guidance, psychological support, sleep optimization, medication management, and management of pregnancy-related complications. The detailed themes regarding needs, satisfaction points, pain points, emotions, and improvement opportunities extracted from the interviews, corresponding to the five stages identified in fieldwork observation, are presented in Table 4.

**Table 4:** Qualitative interview results on prenatal care for rural women.

Stage	Dimension	Descriptions (Simplified)
Establishing medical records	Needs	1. Many matters are involved in the first prenatal examination and time is limited; I hope to complete it quickly 2. No experience with the first child; need professional advice applicable to the whole family
	Satisfaction points	1. Free folic acid provided with instructions on usage 2. Prenatal examinations and infectious disease screenings are covered by medical insurance, with little out-of-pocket expense
	Pain points	1. The process is not posted, leading to unnecessary back-and-forth trips 2. No methods to relieve morning sickness/dizziness/insomnia; having to endure
	Emotions	1. Long waiting time and chaotic process lead to poor experience 2. Professional terms not understood and consultation time is short
	Improvement opportunities	1. Post processes, assign guides, and mark prenatal examination information in handbooks 2. Distribute discomfort relief handbooks and set up pregnancy classes 3. Popularize knowledge via WeChat groups and expert lectures
Non-invasive prenatal testing	Needs	1. Hope to understand the purpose of screening and handling methods for abnormal results 2. Need knowledge on weight control, breast care, etc. during pregnancy
	Satisfaction points	1. Specialized clinics with no crowding and smooth process 2. Doctors explain patiently with illustrations to assist understanding
	Pain points	1. Report professional terms are difficult to understand; doctors do not explain in detail 2. Lack of knowledge on mid-pregnancy nutrition and discomfort relief 3. Unaware of the existence of prenatal schools or unable to understand courses
	Emotions	1. Clear process, short examination time, and good communication experience 2. Reasonable service arrangement at this stage, with the highest satisfaction
	Improvement opportunities	1. Explain reports in simple terms and allow family members to accompany during lectures 2. Popularize knowledge in groups by gestational age 3. Check-in points and family participation for rewards
Ultrasound screening for fetal anomalies	Needs	1. Rely on four-dimensional ultrasound to confirm fetal organ and limb development 2. Need guidance on diet and exercise 3. Want to learn fetal movement counting methods
	Satisfaction points	1. Clear imaging of four-dimensional ultrasound, allowing intuitive observation of fetal face and limbs 2. Doctors explain fetal activity in real-time during the examination
	Pain points	1. No scientific advice on pregnancy medication, diet control, and sleep improvement 2. Short prenatal consultation time; needs of pregnant women with twins not met
	Emotions	1. Not informed of examination precautions in advance; fetal non-cooperation due to fasting 2. Long and tedious examination process; healthcare providers do not take the initiative to guide
	Improvement opportunities	1. Notify examination precautions and time in advance 2. Establish communication platforms for pregnant women with similar gestational ages 3. Distribute illustrated recipe books and life taboo handbooks
Screening for complications	Needs	1. Have family medical history; need screening and prevention guidance for diabetes and hypertension 2. Need breastfeeding guidance such as nipple correction and prevention of milk stasis
	Satisfaction points	1. Color-coded labels in health handbooks to distinguish risk levels with regular follow-up 2. Establishment of family medical history risk management files
	Pain points	1. Glucose screening requires multiple blood draws; long fasting waiting time 2. Unable to use blood glucose meters/blood pressure monitors and unclear about monitoring standards
	Emotions	1. Fasting waiting and long examination time lead to physical discomfort and emotional anxiety 2. Low efficiency of examination after taking leave, resulting in exhaustion of patience
	Improvement opportunities	1. Play popular science videos and conduct short lectures during waiting 2. Add practical training on monitoring tools and breastfeeding simulation training 3. Allow family members to accompany during glucose screening and provide simple supplements
Fetal monitoring/prenatal preparation	Needs	1. Need guidance on the use of home fetal heart rate monitoring tools and data recording 2. Need guidance on childbirth process and prenatal preparation 3. Need methods for emotional regulation and psychological counseling
	Satisfaction points	1. Nurses adjust comfortable positions in advance and explain the process to relieve tension 2. Patiently guide to promote fetal cooperation when the fetus is uncooperative
	Pain points	1. No planning of childbirth routes, inability to use force, and chaotic preparation of prenatal items 2. Advanced age/history of miscarriage; worry about preterm birth but unable to identify signs 3. Improper operation of fetal heart rate monitors and chaotic recording
	Emotions	1. Establish a good relationship with nurses, smooth communication, and anticipation for the baby’s birth 2. Real-time communication about fetal movement during monitoring, leading to a sense of reassurance
	Improvement opportunities	1. Offer training courses on childbirth breathing and pushing techniques 2. Special personnel explain hospital procedures and privacy protection 3. Organize pregnant women communication activities with professional doctors to answer questions

### 3.3 Describing the journey of prenatal healthcare services for rural women

**Figure 1:**
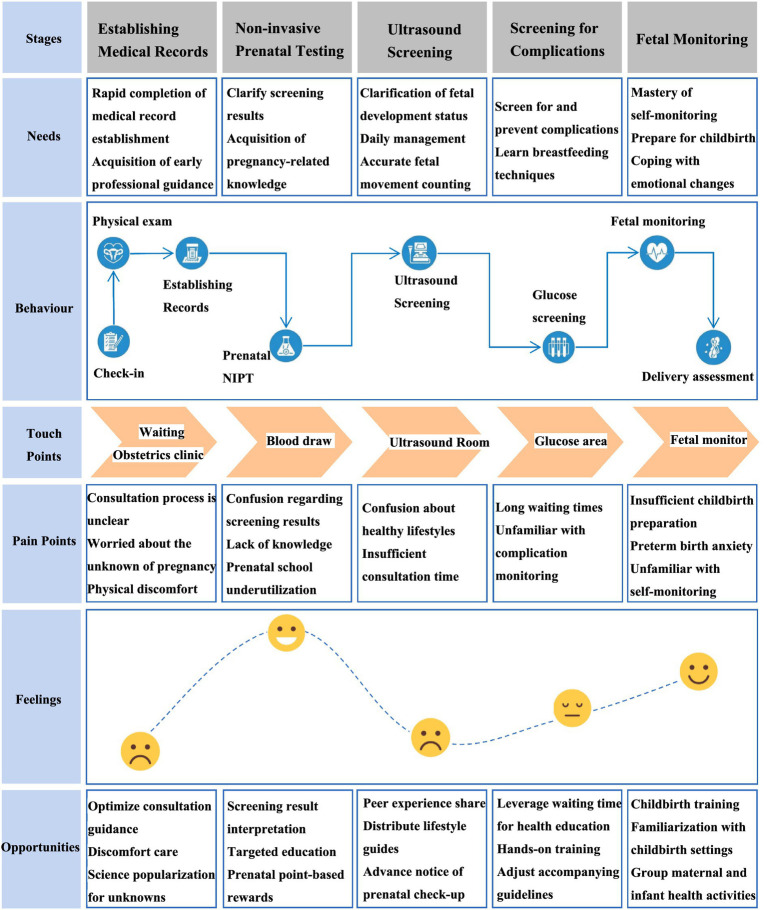
Journey of prenatal healthcare services for rural women.

The five phases of the journey in prenatal healthcare services that were identified are shown in Figure 1.

The map identifies five sequential care stages, core service touchpoints, and key pain points in the current prenatal healthcare system.

## 4 Discussion

This study used a qualitative multi-method design integrating field observation and phenomenological interviews to present the findings in the form of a journey map. The journey map delineates five distinct stages of prenatal healthcare services for rural women, emphasizing key touchpoints, specific needs, and challenges at each stage. This approach can significantly aid healthcare providers in understanding the requirements of pregnant women and developing targeted health management strategies for each phase.

This study aligns with existing qualitative research on prenatal care experiences and extends the literature on maternal health service utilization in rural China. Consistent with prior studies, our findings confirm that rural pregnant women face procedural confusion, physical discomfort, and uneven access to health education. While previous research has identified general barriers, this study provides a more detailed, stage-specific understanding by using journey mapping, which has rarely been applied in the rural Chinese prenatal care context.

Phase 1 marks the initial stage of prenatal care for rural pregnant women. During this phase, they are required to undergo their first prenatal examination, establish a comprehensive prenatal health record, and receive the Mother and Child Health Handbook. Since this stage is the first time for pregnant women to visit medical and health institutions to participate in prenatal care, most of the women interviewed expressed that they were not clear about the departments for registration and the visiting procedures, and they were not familiar with the locations of each examination room either. They could not reasonably plan and arrange the examination sequence, which brought them unpleasant experiences. This was also reflected in the survey. During early pregnancy, fluctuations in hormone levels and increased organ functional load can lead to a variety of physical discomfort symptoms. These symptoms often exhibit synergistic and reinforcing effects, adversely impacting the emotional well-being and quality of life of pregnant women. Nausea and vomiting are the most common early pregnancy discomforts (3). Morning sickness not only impairs nutrient intake but, in severe cases, may compromise fetal development (19). The incidence of pain during early pregnancy is 69.6%, which not only affects maternal mental health but also exhibits a significant linear dose–response relationship with postpartum depression (20). Furthermore, in response to the role transitions, some women also expressed concerns about their future lives during pregnancy. During this stage, based on the actual needs of pregnant women, healthcare institutions may enhance their signage to assist pregnant women in accurately registering and navigating medical procedures. Examination forms may clearly indicate the locations of examination rooms and provide specific recommendations for the sequence of tests. This may help pregnant women streamline the examination process and complete required tests more efficiently. Additionally, group-based health care activities targeting early pregnancy may be implemented to empower pregnant women and enhance their competence in maternal roles (21, 22). These activities may focus on managing physical discomfort and regulating emotional fluctuations during early pregnancy. Meanwhile, it may be beneficial to continue standardizing and promoting basic security services, such as free folic acid distribution with clear usage guidelines and medical insurance coverage for prenatal examinations and infectious disease screenings, to consolidate the existing service advantages.

In the second stage, as depicted in the journey map, pregnant women primarily undergo non-invasive prenatal testing (NIPT). The data indicates that satisfaction with prenatal care peaks during this phase. This high level of satisfaction can be attributed to two key factors. First, while NIPT is generally more expensive, many provinces and regions in China are piloting free NIPT programs for eligible pregnant women, thereby reducing financial barriers. Second, numerous county-level and municipal maternal and child health hospitals have established specialized NIPT clinics staffed by dedicated healthcare professionals, ensuring that pregnant women receive targeted and professional guidance and information. Despite the high satisfaction levels with prenatal care during this stage, several pain points remain. NIPT results typically take 1–2 weeks to be processed. The prolonged waiting period can cause significant anxiety among pregnant women due to uncertainty about the screening outcomes, which tends to increase as time progresses. During the interviews, the majority of pregnant women reported encountering challenges related to weight management and experiencing confusion regarding proper nutritional intake during this stage. The global prevalence of overweight and obesity during pregnancy is increasing (23). Women with a body mass index (BMI) greater than 25 kg/m^2^ are more likely to experience difficulties in conceiving and have an increased risk of miscarriage and stillbirth compared to those within the ideal BMI range (24). Additionally, all pregnancy complications are more prevalent among women who are overweight or obese, or who experience excessive gestational weight gain, including conditions that pose a significant threat to maternal and neonatal health (25). Traditional health education often focuses on providing recommendations for types and quantities of nutrients. However, pregnant women face numerous challenges in translating these guidelines into practical daily diets, as it is difficult to quantify and apply them effectively. To address these concerns, targeted health education activities may be implemented during this period, focusing on themes that align with the primary needs and concerns of pregnant women. To support better nutrition and weight management, information may be presented in a clear and detailed recipe manual, accompanied by recommended pregnancy weight gain standards. This approach may enable pregnant women and their families to more intuitively understand and practice appropriate dietary and weight management strategies during pregnancy.

Ultrasound screening is a critical examination in Stage 3 and an essential component of prenatal care. Detailed ultrasound screening allows for the assessment of fetal development and the detection of potential congenital anomalies (23). Consequently, rural pregnant women often opt for higher-level municipal medical institutions, despite their greater distance, which incurs additional transportation costs and time. However, satisfaction levels among pregnant women during this stage are relatively low. The primary reason is that many women lack clear guidance on how to effectively cooperate with healthcare providers to ensure smooth and successful examinations, leading to repeated visits to the ultrasound room or the need for rescheduled appointments. During this stage, pregnant women may experience changes such as insomnia and frequent urination, which can significantly impact their daily lives (26, 27). Insufficient sleep during pregnancy is associated with an increased risk of adverse outcomes, including premature birth, cesarean section, gestational hypertension, gestational diabetes, and prolonged labor (28). Additionally, it is a significant risk factor for maternal depression, internalizing mental health issues in children, and symptoms of attention-deficit/hyperactivity disorder (ADHD). However, it is important to note that many rural pregnant women report that distance and time constraints prevent them from actively participating in activities such as prenatal schools, which significantly limits their utilization of available resources. To improve examination experience and efficiency, healthcare providers may provide detailed guidance on examination procedures, purposes, and cooperative strategies after each consultation. This may include advice on scheduling appointments during periods of fetal activity, wearing comfortable clothing, eating lightly, and using gentle methods such as abdominal massage or music to stimulate appropriate fetal position and movement. These measures may help improve examination efficiency and reduce the need for repeated visits. The focus of prenatal care during this stage may be placed on supporting coping strategies for physical discomfort and fetal movement monitoring, with the aim of enhancing pregnant women’s self-management capabilities and encouraging healthy daily routines. To improve access to health education, online platforms may be used to deliver learning resources, and incentive mechanisms such as point systems may be adopted to encourage timely participation (29). Furthermore, peer support among pregnant women at similar gestational stages may be encouraged to facilitate information sharing and mutual assistance (30).

In Stage 4 of prenatal care, healthcare services primarily concentrate on the prevention and screening of pregnancy-related complications. During this phase, it is crucial for pregnant women to learn how to monitor their daily blood pressure and glucose levels and to undergo the glucose tolerance test (GTT) (31). Unlike other routine examinations, the GTT is a prolonged process that involves multiple venous blood draws over a specified period. Due to delayed appointment scheduling, many women experience significant hunger during the waiting period. Additionally, the unpalatable taste of the concentrated glucose solution often leads to nausea and vomiting in numerous cases. These factors contribute significantly to lower satisfaction levels among pregnant women regarding prenatal care services during this stage. Furthermore, it is important to highlight that rural pregnant women in China are influenced by traditional family child-rearing practices and economic constraints. Consequently, most rural pregnant women do not opt for professional postpartum care services such as maternity centers or nannies. Instead, the primary caregivers for both the mother and newborn are typically the mothers of the couple. However, traditional parenting beliefs often fail to meet the needs of modern pregnant women. To address the discomfort associated with the GTT, adjustments may be made to the glucose solution—such as modifying its temperature or adding appropriate, non-interfering additives—to improve taste and reduce nausea (32). Additionally, the two-hour waiting period may be utilized for targeted health education to divert pregnant women’s attention. This health education may include hands-on simulation exercises for blood pressure measurement and daily blood glucose monitoring, encouraging pregnant women’s active participation in practical learning. To address the gap in postpartum care knowledge, advance education may be provided to rural pregnant women and their family members, focusing on proper childcare methods, correct breastfeeding techniques, and appropriate postpartum care (33, 34).

The final stage of prenatal care services spans from 32 weeks of gestation until delivery. During this period, pregnant women require regular and repeated fetal heart rate monitoring to assess the intrauterine development of the fetus. Compared with earlier stages, women in this phase utilize prenatal care services more frequently and engage in closer communication with healthcare providers. This increased interaction offers greater opportunities for enhancing the quality of prenatal care services. During the final trimester of pregnancy, women experience a significant increase in physical discomfort. Common symptoms such as pain, edema, insomnia, frequent urination, and gastrointestinal distress frequently arise (35, 36). Coupled with the anticipation and anxiety surrounding impending childbirth, pregnant women have an increased need for emotional support from family members and professional guidance from healthcare providers (37). Physical limitations in the third trimester may also lead to lower participation rates in offline prenatal health care activities, particularly among rural pregnant women living far from medical facilities. To improve accessibility and engagement, online platforms may be used to deliver tailored educational content to pregnant women and their primary caregivers. Healthcare providers may guide pregnant women in managing late-pregnancy discomforts and recognizing signs of labor, helping ensure they receive timely and appropriate information. In addition, routine prenatal visits may be fully utilized to familiarize women and their families with the delivery environment and procedures. Healthcare providers may assist in developing a comprehensive transition plan, including transportation routes, delivery companions, and preparation of maternity items for hospital admission.

## 5 Study limitations

This study has several limitations that should be acknowledged. The study was conducted in two cities in Northeast China; thus, findings may not be generalizable to other rural areas in China. First, the study population was restricted to rural pregnant women receiving prenatal care in Anda City and Daqing City, Heilongjiang Province, Northeast China. The socioeconomic conditions, cultural norms, and access to maternal and child health services in this region may differ from those in other rural areas of China, which limits the generalizability of the findings to the broader rural pregnant population nationwide. Second, the qualitative interview sample size was relatively small (*n* = 16), although data saturation was achieved through purposive sampling covering diverse demographic characteristics. A larger and more geographically diverse sample could enhance the robustness and representativeness of the identified needs and pain points. Third, observer effects may have been present during field observation, as the presence of researchers could have influenced the behaviors of pregnant women and healthcare providers, despite efforts to maintain a non-intrusive stance. Fourth, interview data were affected by potential recall bias and social desirability bias, as participants may have retrospectively reconstructed experiences or provided responses they perceived as more socially acceptable. Fifth, purposive sampling was adopted to recruit participants with diverse characteristics, which may limit the transferability of the findings to other rural settings with different health system structures and cultural contexts. These limitations do not undermine the practical value of the study’s findings, as the constructed journey map still provides actionable insights for optimizing prenatal care services for rural women in similar socioeconomic and healthcare contexts. Instead, they highlight important directions for future research to address the gaps and enhance the comprehensiveness and generalizability of related studies.

## 6 Conclusion

This exploratory qualitative study conducted in rural Northeast China constructed a five-stage journey map of prenatal healthcare service utilization, which visually and systematically illustrates the experiences, perceptions, and needs of rural pregnant women at each stage. These findings are context-specific to rural Northeast China and provide hypothesis-generating insights for similar regions. Caution should be taken in generalizing the results to broader rural populations across China. The journey map can help identify key improvement points of prenatal care services, enhance women’s self-management capabilities, and support the targeted optimization of prenatal healthcare management for rural women in comparable settings.

## Data Availability

The raw data supporting the conclusions of this article will be made available by the authors, without undue reservation.
