# Supplementary material for: A qualitative journey mapping study of prenatal care experiences among rural pregnant women in Northeast China
Source: Front Public Health. 2026 Apr 29;14:1787403. doi: 10.3389/fpubh.2026.1787403 (PMC13168180; doi:10.3389/fpubh.2026.1787403)
Supplement: Supplementary file 1 [file Table_1.DOC]

| **Stage** | **Participants** | **Objects** | **Activities** |
| --- | --- | --- | --- |
| Establishing Medical Records for Pregnant Women | Pregnant women  Family members  Obstetric doctor  Ultrasound physician  Obstetric nurse  Phlebotomy nurse | Waiting area  Signage  Electronic display  Bulletin board  Mother and child health handbook | Sign-in  Medical consultation  Medical examination  Feedback on examination results |
| Non-invasive Prenatal Testing | Pregnant women  Family members  Obstetric doctor  Phlebotomy nurse | Waiting area  Phlebotomy Room  Ultrasound Room | Sign-in  Medical consultation  Medical examination  Feedback on examination results(≥2weeks) |
| Ultrasound Screening | Pregnant women  Family members  Obstetric doctor  Ultrasound physician  Obstetric nurse | Waiting area  Ultrasound Room  Music playback system  Food | Sign-in  Medical consultation  Medical examination  Feedback on examination results |
| Screening for Complications | Pregnant women  Family members  Obstetric doctor  Obstetric nurse  Phlebotomy nurse | Waiting area  Water glass  Glucose  Glucose administration area  Phlebotomy Room  Sphygmomanometer  Body weight scale | Sign-in  Medical consultation  Medical examination(≥2h)  Feedback on examination results |
| Fetal Monitoring/Prenatal Preparation | Pregnant women  Family members  Obstetric doctor  Obstetric nurse | Waiting area  Fetal heart monitor  Belt  Music playback system  Food  Sphygmomanometer  Body weight scale  Soft ruler | Sign-in  Medical consultation  Medical examination(≥40min)  Feedback on examination results |

**Table S1** Detailed Settings, Participants and Activities of Prenatal Care Stages
